# Plasmonic eigenmodes in individual and bow-tie graphene nanotriangles

**DOI:** 10.1038/srep09535

**Published:** 2015-04-09

**Authors:** Weihua Wang, Thomas Christensen, Antti-Pekka Jauho, Kristian S. Thygesen, Martijn Wubs, N. Asger Mortensen

**Affiliations:** 1 Center for Nanostructured Graphene (CNG), Technical University of Denmark, DK-2800 Kongens Lyngby, Denmark; 2 Department of Photonics Engineering, Technical University of Denmark, DK-2800 Kongens Lyngby, Denmark; 3 Department of Micro and Nanotechnology, Technical University of Denmark, DK-2800 Kongens Lyngby, Denmark; 4 Department of Physics, Technical University of Denmark, DK-2800 Kongens Lyngby, Denmark

## Abstract

In classical electrodynamics, nanostructured graphene is commonly modeled by the computationally demanding problem of a three-dimensional conducting film of atomic-scale thickness. Here, we propose an efficient alternative two-dimensional electrostatic approach where all calculation procedures are restricted to the graphene sheet. Furthermore, to explore possible quantum effects, we perform tight-binding calculations, adopting a random-phase approximation. We investigate multiple plasmon modes in 20 nm equilateral triangles of graphene, treating the optical response classically as well as quantum mechanically. Compared to the classical plasmonic spectrum which is “blind” to the edge termination, we find that the quantum plasmon frequencies exhibit blueshifts in the case of armchair edge termination of the underlying atomic lattice, while redshifts are found for zigzag edges. Furthermore, we find spectral features in the zigzag case which are associated with electronic edge states not present for armchair termination. Merging pairs of triangles into dimers, plasmon hybridization leads to energy splitting that appears strongest in classical calculations while splitting is lower for armchair edges and even more reduced for zigzag edges. Our various results illustrate a surprising phenomenon: Even 20 nm large graphene structures clearly exhibit quantum plasmonic features due to atomic-scale details in the edge termination.

The collective excitations of conduction electrons in noble metals have been of great interest for a very long time. These excitations known as plasmons play an important role in the optical properties of metals. Through strong plasmon-photon interactions, metals can support important phenomena, such as focusing beyond the diffraction limit^1^, squeezing the light down to nanoscale^2^, and large local field enhancement^3^. Due to these features, plasmons in metals give rise to various potential applications, and especially form a bridge between the worlds of photonics and electronics which commonly work at different length scales^4^. Developments in nanofabrication technology have stimulated a series of plasmon-based devices like waveguides^5^, filters^6^, switches^7^, and modulators^8^. In many respects, plasmonic devices open a door to a better performance in speed and size, holding potential for faster dynamics than electronic devices while still having a smaller size footprint than the common all-dielectric photonic devices. However, the inherent Joule loss in metals severely hampers many practical applications of plasmonics^9^. Alternatively, attempts have already been made to study plasmonics in materials other than metals^10^, for example doped semiconductors^11^ and superconductors^12^,^13^.

Graphene and other low-dimensional crystals are now emerging as interesting materials for exciting science and technology^14^. Here we study the plasmonic properties of graphene flakes. In its pristine form graphene is a semimetal, but with appropriate doping it is emerging as a promising plasmonic material as well^15^,^16^,^17^,^18^,^19^. The graphene plasmons are non-radiating, but with a momentum mismatch to free-space radiation that can be overcome with the aid of e.g. grating approaches^20^. The charge carriers in graphene obey linear energy dispersion at lower energies close to the Dirac points, thus resembling the linear dispersion of photons^21^,^22^,^23^. Experimental investigations of carrier transport show that the mobility limited by impurity scattering can exceed 15.000 cm^2^/Vs at room temperature^21^, which gives the intrinsic loss in graphene one order of magnitude less than the noble metals. Despite relaxation due to phonon scattering^24^,^25^, graphene achieves superior plasmonic performance in propagation length and field enhancement^26^,^27^. The carrier density in graphene may be adjusted by electrostatic gating, which results in actively tunable plasmons beyond structural variations in metals, as has already been demonstrated experimentally^28^,^29^. With the typical doping levels, the plasmonic response is generally in the terahertz (THz) to mid-infrared frequency range, thus allowing new progress in THz technology^30^. As an example, graphene waveguides (with sub-wavelength width) pave a promising way to realize ultra-compact THz devices where bends and splitters do not bring any significant loss^31^.

Because of these attractive plasmonic properties, it is worth to comprehensively study the optical properties of graphene. Here the fundamental quantity is the dielectric function. For graphene systems, the dielectric function can be obtained within the framework of linear-response theory and the random-phase approximation (RPA)^15^,^16^,^32^. For infinite graphene sheets, the derived two-dimensional (2D) dielectric function *ε*(*q*,*ω*) is a function of both frequency *ω* and momentum *q*. This is different from common three-dimensional (3D) photonic materials which are usually well-described by frequency-dependent functions, while spatial dispersion is negligible for good dielectrics and most metals (beyond the nanoscale). Two common approximations in the modelling of graphene structures are to adopt the local-response approximation (applying the small-*q* limit) and to model graphene as a very thin conducting film, yet preserving its 3D representation^33^,^34^. Using dielectric functions so obtained, one can solve Maxwell's equations for arbitrarily shaped flakes of nanostructured graphene. For very small flakes of characteristic dimension *R* (*R* ~ *λ*_F_ with *λ*_F_ ~ 10 nm being the Fermi wavelength corresponding to a Fermi level of 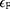 = 0.4 eV), the common assumption 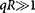 is jeopardized and nonlocal response turns important for far-field optical properties. Obviously, near-field properties may be influenced too, e.g. for dimers of sufficiently large structures, where 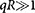 holds, while instead the tiny gap (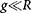) promotes nonlocal effects^35^. For optical excitation in the near field^17^,^36^, nonlocal response can also be important for large plasmonic structures provided that the distance to the emitter is comparable to the nonlocal characteristic length scale^37^. In this regime, both semiclassical hydrodynamic^38^,^39^ and full quantum approaches have been proposed^40^,^41^, similar to those recently developed for metals^35^,^42^. While previous studies have mainly focused on the optically bright dipole mode, here we will illustrate that structured graphene is also rich on higher-order modes. Although the latter are typically not excited by far-field radiation, they may be probed by near-field optical spectroscopy and/or electron energy loss spectroscopy (EELS).

In this article, we study plasmon properties in individual graphene nanostructures and in dimers of such structures by means of both classical and quantum methods. In particular, we consider triangles of graphene and bow-tie structures formed by such triangles, while our methods can also be applied to other geometries (as we show in the Supplementary Information). Here, we focus on the plasmonic aspects due to doping with a significant number of electrons, while there are also appealing aspects in the single-electron doping regime^43^. We will emphasize dimers formed by electrically mutually disconnected graphene islands, while graphene dimers connected by quantum junctions^44^ and extended complimentary structures (electronic^45^ and plasmonic^25^,^46^ periodic anti-dot arrays) also represent interesting geometries and regimes. As a key element, we consider the eigenmodal properties of the plasmonic excitations, thus extending the current state of analysis beyond dipolar excitations, as relevant for near-field plasmonic interaction with e.g. emitters or fast electrons.

In our classical electrodynamical considerations, we treat the nanostructures as 2D materials characterized by a smooth surface conductivity (employing the sheet conductivity derived for bulk graphene), and formulate a closed-form eigenvalue problem on a 2D domain. Numerical solutions in arbitrarily shaped geometries are enabled by finite-element calculations. By its nature, this classical approach neglects the atomic details of the graphene flake. Some aspects e.g. of zigzag termination can be effectively accounted for by additional conductive channels^39^, though we will not pursue this scheme for our classical calculations presently.

In our quantum treatment, we employ a tight-binding description^40^,^41^ to account for the actual position of all atoms in the flake and in particular the edge atoms which have the possibility for either armchair or zigzag configurations (Other edge structures can arise from the mixture of these two configurations, but they will not be discussed here). In both the classical and the quantum calculations, multiple plasmon modes are extracted including dipole, multipole, and breathing modes. Their hybridized counterparts in bow-tie nanostructures are also discussed. We show that plasmon excitations and hybridizations are extremely sensitive to the electronic edge effects. This illustrates how quantum plasmonics can manifest itself in graphene structures with dimensions much exceeding the length scales for nonlocal response in individual noble-metal nanoparticles^35^.

## Results

### Classical Description

Modern computational electromagnetics is commonly optimized to explore the interaction of radiation with matter in a three-dimensional space, so that two-dimensional material problems are typically not efficiently addressed with existing numerical schemes. For example, a pragmatic approach is to simply mimic the atomically thin graphene layer with a homogenous dielectric film of a finite, yet small thickness *t*_g_. This assumed 3D film has an effective bulk permittivity, *ε*(*ω*) = *ε*_0_ + *iσ*(*ω*)/(*ωt*_g_), where *ε*_0_ is the vacuum permability and *σ*(*ω*) denotes the surface conductivity as obtained from e.g. the local-response limit of the RPA^33^,^34^. Evidently, the artificial thickness *t*_g_ should be chosen sufficiently small compared with all other characteristic and physical dimensions, yet sufficiently large that meshing hopefully stays computationally feasible and the numerical problem remains tractable. Optimizing this thickness tradeoff does not necessarily give an efficient method. An even more critical issue is that there are no formal proofs, at least to the best of our knowledge, that numerically computed fields (in particular near fields relevant for LDOS or emitter dynamics near a graphene structure^17^,^36^) would necessarily converge to physically meaningful quantities in the limit *t*_g_ → 0. Alternatively, in nanostructures with high symmetry, e.g. in ribbons^47^,^48^ or disks^39^,^49^, one may take advantage of modal expansion methods^39^,^47^,^48^,^49^ – which, however, is not an appealing choice for more general structures, where limited analytical progress is possible. In the following, we develop a 2D finite-element approach to efficiently solve the electromagnetic problem self-consistently for graphene in terms of the electric potential and induced charge in general structural configurations.

With the typical sub-eV doping levels, plasmonic resonances typically occur in the mid-infrared regime. The associated free-space wavelength (~10 *μ*m) is then much larger than the geometrical extent of the hosting graphene nanostructures (~10–100 nm). For such problems the electrostatic approximation is excellent. As a computationally very attractive consequence, the electric and constitutive response are governed by two coupled scalar equations for the potential *φ* and the induced density *ρ*. In particular, we note that the total potential *φ*(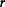) is governed by Coulomb's law
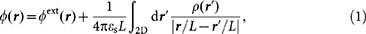
where *φ*^ext^(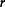) denotes the external potential, *L* is an auxiliary quantity such as the feature length of the structure which makes the surface integral dimensionless, *ρ*(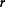
′) the induced surface charge density, and *ε*_s_ = (*ε*_above_ + *ε*_below_)/2 the averaged dielectric constant of the medium above and below graphene. For simplicity, we only consider freely suspended graphene, so we will use *ε*_s_ = *ε*_0_ throughout the remaining part of the paper. The other scalar equation is obtained by inserting the constitutive equation ***J***_2D_ = −*σ*(*ω*)∇_2D_*φ*(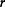) into the continuity equation *iωρ*(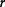) = ∇_2D_·***J***_2D_(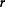), which for ***r*** restricted to the plane of the graphene structure gives 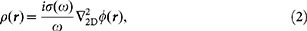
with 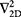 the 2D Laplace operator. Equation (2) is solved subject to the assumption of charge neutrality, i.e. 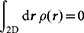, implying that 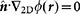 on the boundary of the domain, with 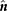 denoting the in-plane surface normal. The density *ρ* in (2) is restricted to the graphene plane. It may be obtained from a closed-form equation by eliminating the potential in (2) with the help of (1) (see Methods for additional details)^50^. Once *ρ* within the graphene plane is thus obtained, the potential *φ* in the entire space can be evaluated via (1).

Within the framework of the finite-element method (FEM), Eqs. (1) and (2) can both be recast as matrix equations. Concretely, by denoting the FEM-discretized potentials and induced charge densities by vectors, we find the equations ***φ*** = ***φ***^ext^ + (4*πε_s_L*)^−1^**A*ρ*** and ***ρ*** = *iσ*(*ω*)*ω*^−1^**B***φ*, which we combine to get
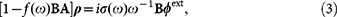
where **A** and **B** are geometry-dependent square matrices, respectively representing the Coulomb integral in Eq. (1) and the Laplacian in Eq. (2) (see the Methods section below for additional details), while *f*(*ω*) = *iσ*(*ω*)/(4*πε_s_Lω*) is a geometry-independent scalar^29^. The term in the square brackets on the left-hand side of Eq. (3) represents the matrix-form of the effective (classical) frequency-dependent dielectric function ***ε***^CLA^(*ω*) ≡ **1** − *f*(*ω*)**BA**, connecting total and external potentials via ***ε***^CLA^(*ω*)***φ*** = ***φ***^ext^. In the absence of an external potential (***φ***^ext^ = 0), Eq. (3) becomes an eigenvalue problem for the matrix **BA**. The resulting eigenvalues *λ_n_* are associated with plasmon frequencies *ω_n_* through 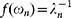, and the associated eigenvectors are induced charge densities ***ρ****_n_* in a finite-element representation. The corresponding eigenpotentials are denoted as ***φ****_n_*, and within the graphene plane they can be computed directly as ***φ****_n_* = **A*ρ****_n_*. Following this classical approach, all plasmonic eigenmodes for a specific structure can be obtained as the solution of a single eigenvalue problem. This constitutes an attractive computational approach that can give direct insight in the plasmonic eigenstates that one would be able to probe with various experimental techniques.

### Quantum Mechanical Tight-Binding Description

In a quantum mechanical formalism, there are two key computational components: (i) electronic band structure, and (ii) determination of response functions. The graphene *π* and π* bands (valence and conduction bands respectively) originating from the carbon 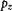 orbitals are well separated in energy from the four *σ* bands arising from *sp*^2^ hybridization. The dynamics of low-energy excitations in graphene is well-described by inclusion of just the *π* bands, which can be determined by a simple tight-binding model in a nearest-neighbor approximation^51^,^52^. Specifically, a graphene nanostructure with *N* carbon atoms results in an *N* × *N* matrix representation of the tight-binding Hamiltonian with elements determined by the 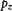 orbital hopping integral. A direct diagonalization of the Hamiltonian yields *N* eigenvalues and eigenvectors, corresponding to the electronic energy levels and the wave functions, respectively. The non-interacting density response function, or polarizability matrix 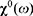, is then built from the electronic states whose elements are given by^15^,^16^,^32^
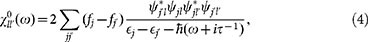
where 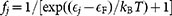 denotes the Fermi–Dirac distribution function associated with the state with energy 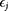 and wave function 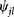 (*l* labels each of the carbon atoms), while *k*_B_ and 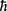 are Boltzmann's and Planck's constants, respectively. The factor 2 accounts for spin degeneracy in the absence of a static magnetic field with no Zeeman splitting. In both classical (also called semi-classical due to the conductivity including Fermi–Dirac distribution function) and quantum calculations, states are populated in accordance with a Fermi level of 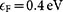 and a temperature *T* = 300 K corresponding to a thermal energy of 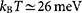. We phenomenologically account for scattering losses through a relaxation time 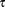 corresponding to 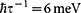, commensurate with experimental data at the considered doping level^53^. Naturally, resonances are influenced by both the doping level (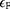), the relaxation time (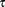), the dielectric substrate properties (*ε*_s_), and the characteristic structure dimensions (*L*). For details, we refer to the Supplementary Information.

In the following, we use an efficient method to compute the non-interacting density response matrix 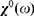, based on Hilbert and fast Fourier transforms (see Ref. 40 and Methods section below). Including the effects of a self-consistent Hartree interaction, i.e. within the RPA, the interacting polarizability is given by^32^
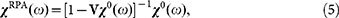
with the Coulomb interaction 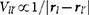 for *l* ≠ *l*′, and a self-interaction of 0.58 atomic units at *l* = *l*′^40^. The poles of 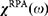 or equivalently the zeros of the denominator
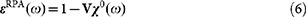
give the plasmon frequencies. More accurately, since ***ε***^RPA^(*ω*) is a matrix, we in principle seek the eigenvalues *ε_n_*(*ω*) of the ***ε***^RPA^(*ω*) whose real part approaches zero^54^. In practice there is also loss, for example due to 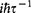 in 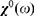. The eigenfrequencies *ε_n_*(*ω*) are therefore complex-valued, with the imaginary parts denoting the plasmon peak broadening. With this in mind, we finally define the plasmon resonance frequencies from the local maxima of 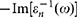
54,55.

Numerically, the eigenvalues *ε_n_*(*ω*) are obtained by diagonalizing the RPA dielectric function ***ε***^RPA^(*ω*) for each frequency. An *N*-atom nanostructure entails *n* = 1,2,3, …, *N* distinct eigenvalues. Out of these we focus in the following on eigenvalues with largest and second-largest values of 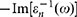. Their corresponding eigenvectors are the induced charge densities ***ρ****_n_*, and similarly the eigenpotentials ***φ****_n_* can be obtained by performing the Coulomb integral. For comparison with the quantum treatment, we also calculate the eigenvalue loss spectrum in the classical framework by carrying out diagonalization of the classical effective dielectric function ***ε***^CLA^(*ω*).

### Plasmonic Eigenmodes in Individual Triangles

**Figure 1:**
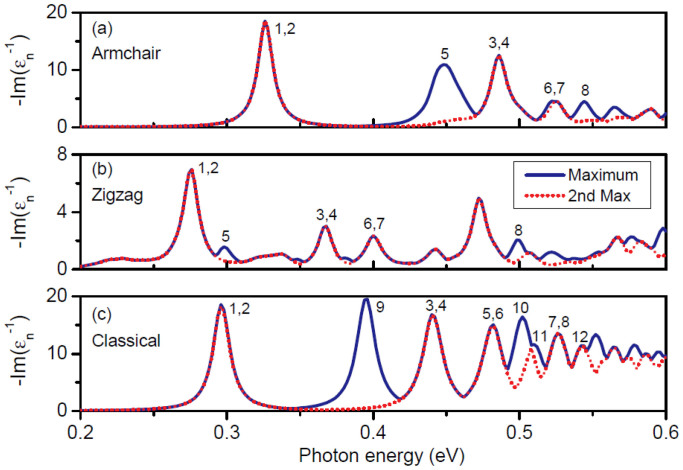
Eigenvalue loss spectrum in graphene triangles. The loss function 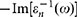 is calculated for equilateral graphene triangles of sidelength 20 nm. Each peak defines a plasmon mode (labeled by *n* = 1,2,3,…), and the coincidence of the maximum (blue solid) and the second maximum (red dotted) indicates the energy degeneracy. Results of the quantum tight-binding method in (a) for armchair edges, and in (b) for zigzag edges, while classical results are given in (c).

**Figure 2:**
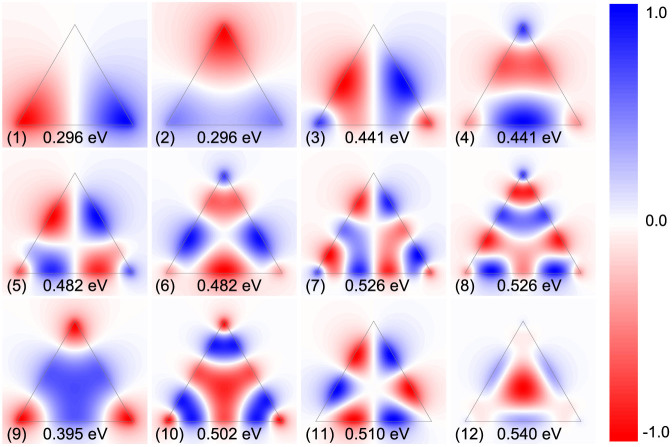
In-plane classical eigenmode potentials. The real part of ***φ****_n_* for the twelve lowest-energy plasmon modes calculated in the classical approach, from the eigenvectors of the matrix pair **BA** of Eq. (3).

The calculated eigenvalue loss spectrum for 20 nm graphene equilateral triangles is shown in Figure 1. In the quantum description we distinguish between zigzag and armchair edge terminations, see Supplementary Information. Multiple plasmon peaks are visible in the considered frequency regime. Additionally, at several frequencies, the two considered loss functions (largest and second largest values of 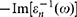) are nearly identical, while at other frequencies one can be resonant while the other one is not. This is in full accordance with group-theoretical considerations for our structure with *m*-fold rotational symmetry where the *C_m_* point group leads to either non-degenerate eigenstates or pairs of eigenstates with a double degeneracy^56^. The degeneracy can be explored further by considering the eigenmodes, expressed e.g. by the in-plane potential, and in particular their symmetries. In the classical approach, the eigenmodes appear as eigenvectors of the matrix **BA** of Eq. (3). Considering the two lowest eigenstates causing the resonance around 0.3 eV in Figure 1(c), we numerically find the eigenfrequencies to be 0.2964 eV and 0.2963 eV. The small energy difference of 0.1 meV illustrates the numerical accuracy (symmetry breaking) associated with the fact that our finite-element mesh does not comply with the threefold rotational symmetry of the graphene triangle. In Figure 2 we show corresponding in-plane potential distributions of the twelve lowest-energy eigenmodes, again calculated in the classical framework. The eigenmodes are responsible for the primary features of Figure 1c; specifically, the loss-function exhibits peaks at the resonance energies of the eigenmodes. The peaks are each assigned a label (*n* = 1,2,3,…), corresponding to the eigenmode enumeration in Figure 2. A one-to-one correspondence is evident and whenever the spectrum in Figure 1 suggests a pair of degenerate states, the corresponding modes in Figure 2 support that they are indeed pairs of orthogonal and degenerate states. The energy degeneracies exhibited here are a direct consequence of the symmetries of the considered nanostructure, as required by group theory^57^.

The plasmon modes 1 through 8, being doubly degenerate, are either symmetric or antisymmetric with respect to the mirror symmetry plane. The dipole modes, 1 and 2, with the electric field being polarized orthogonal to each other, are of particular interest due to their strong coupling to optical fields. They can be excited directly by far-field techniques, and the plasmonic local field enhancement is concentrated at the vertices. The modes 3 through 8 penetrate significantly into the bulk, and can be considered as hybridized modes originating from interaction between dipole and bulk modes, because the patterns at the vertices are similar to dipole modes 1 and 2; in addition, the modes 3–6 have finite net dipole momenta, and can couple to far-field radiation. The modes 9–12 are not doubly degenerate, and exhibit threefold rotational symmetry around the center. Although optically dark, these modes are still detectable by suitable near-field techniques. As an example, in an EELS experiment the breathing mode 12 would exhibit the strongest coupling to a nanometer-sized electron beam if this beam passes through the center of the graphene triangle^58^.

**Figure 3:**
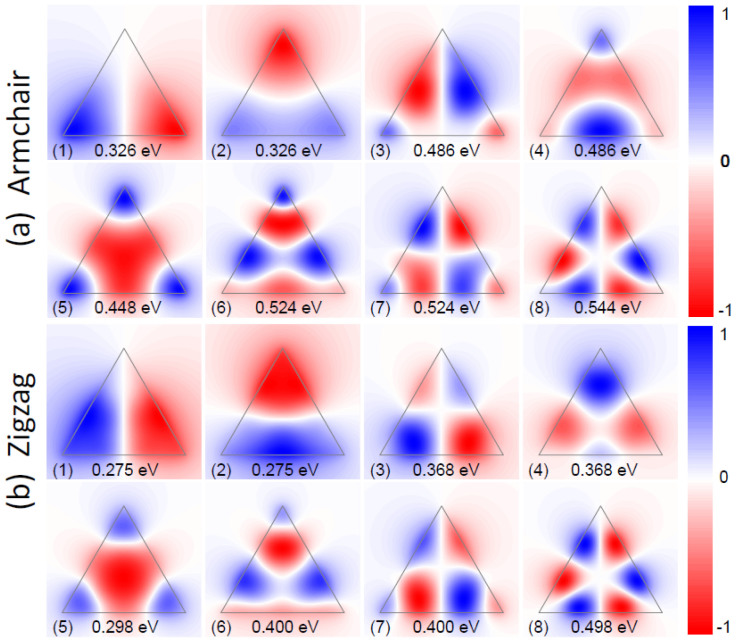
In-plane quantum eigenmode potentials. The real part of ***φ****_n_* as extracted from the eigenvalue loss spectrum calculations. (a) The lowest 8 plasmon modes in an armchair triangle; (b) the corresponding plasmon modes in a zigzag triangle.

Having described our classical results for graphene triangles, let us now turn to our corresponding tight-binding quantum results. In the quantum description, we calculate the eigenvalue loss spectrum, identify the plasmon mode eigenfrequencies, and then extract the corresponding eigenmodes. Due to the geometrical symmetry, the plasmon eigenmodes should exhibit the same energy degeneracy features as the equilateral triangles in classical calculations, for instance in Figure panels 1(a) and 1(b) several doubly degenerate plasmon modes occur. Figure 3 shows the wave patterns from the quantum calculations, corresponding to the peak labeling in Figure 1(a) and 1(b). We observe that for the armchair case the modes of the same type are blueshifted when compared to their classical counterparts. On the contrary, zigzag termination incur lower plasmon energies with a net redshift compared to the classical case. As an concrete example, the eigenfrequencies of the dipole modes are 0.326 eV, 0.275 eV, and 0.296 eV for the armchair, zigzag, and classical cases, respectively. The associated mode patterns are only slightly different, yet it is clearly seen from the dipole modes, that in zigzag-terminated triangles the mode spreads much more into the bulk while for armchair termination the mode concentrates at the vertices in the same manner as for the classical results. This trend becomes even more evident in the modes 3 and 4 of which the patterns show no hot spots at the vertices. The shifts of armchair and zigzag structures relative to the classical results were recently discussed from an analytical perspective^39^, and attributed, essentially, to two effects. For the armchair, a nonlocal blueshift accounts for the observed behavior. In the zigzag case, in addition to a nonlocal interaction, the existence of edge states enables an additional dispersive channel, which leads to a net redshift. Similar edge states do not exist for armchair terminations (see Supplementary Figure S3 for additional details). The role of edge states has previously been examined numerically in graphene ribbons^40^, disks^41^, and triangles^43^.

### Plasmon Hybridization in Bow-Tie Triangles

**Figure 4:**
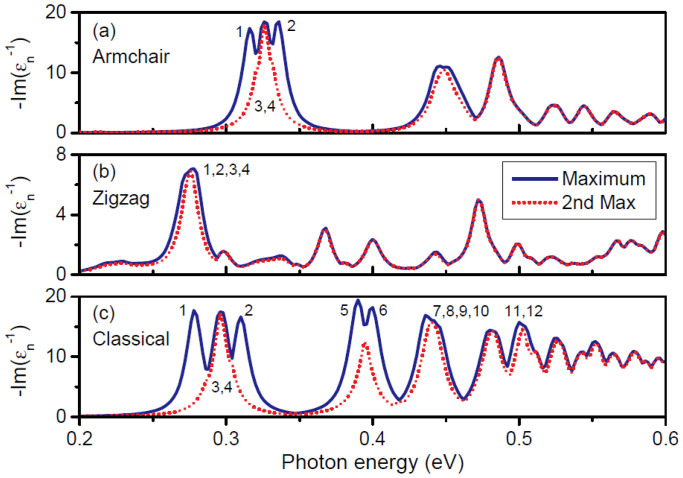
Eigenvalue loss spectrum in graphene dimers. The loss function 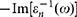 calculated for graphene bow-tie triangles with gap width 0.5 nm. Results obtained from quantum calculations in armchair triangles (a) and in zigzag triangles (b) are compared with classical calculations in (c).

**Figure 5:**
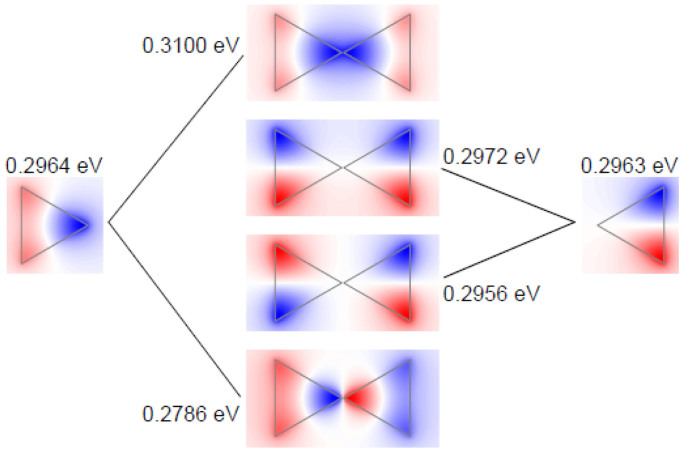
Hybridization of plasmons in graphene dimers. Schematic diagram of the dipole mode hybridization in classical calculations. There is a larger energy splitting for *x*-polarized dipole, and the antisymmetrically coupled modes have lower energy for both polarizations. Here the gap distance is 0.5 nm.

Plasmon hybridization is of both fundamental and practical importance^59^,^60^. Hybridization through tuning of the gap distance can be used to achieve better performance through careful design, such as the field enhancement in dimers^61^ and the sensing capabilities in Fano structures^62^. Here, we study the plasmon hybridization in graphene bow-tie triangles, using the same classical and quantum methods as for individual triangles above. Figure 4 shows the calculated eigenvalue loss spectra for a gap width of 0.5 nm. There are four modes (*n* = 1,2,3,4) in the classical calculations, originating from the four (accounting degeneracy) low-energy dipole modes of the two uncoupled triangles. The hybridization process is illustrated in Figure 5 with a focus on dipole modes, where energies are given with higher precision in order to display the tiny energy shifts associated with the hybridization. We find that each dipole mode in the individual triangles will split into two modes in the bow-tie triangles forming either bonding or antibonding states. The *x*-polarized dipole (0.2964 eV, dipole aligned parallel to bow-tie axis) exhibits large energy splitting, and the corresponding bonding (antisymmetrically coupled) mode has lower energy. However, for the *y*-polarized dipole (0.2963 eV, dipole aligned perpendicular to bow-tie axis) the reduced mode-overlap causes a very small energy splitting. In both cases, the bonding modes are optically active with a net dipole polarization along *x* and *y* direction, respectively.

We find a very similar behavior in the armchair-terminated bow-tie triangles shown in Figure 4a, but with smaller energy splitting, which originates from a weaker mode overlap and weaker coupling strength when compared to the classical calculations. In the zigzag-terminated bow-tie triangles (see Figure 4b), the coupling strength is even weaker and the *x*-polarized dipole exhibits no appreciable energy splitting when compared to the line width of the uncoupled resonances. As a result of this approximate degeneracy, the coupled system exhibits a single broad peak with all four modes merged together. In contrast to the dipole modes, the higher-order plasmon modes show a weak lifting of degeneracy for antisymmetrical and symmetrical states. We mention that the hybridization picture given in Figure 5 is very general, also being satisfied in quantum calculations but with different eigenfrequencies (hybridization diagrams not shown).

**Figure 6:**
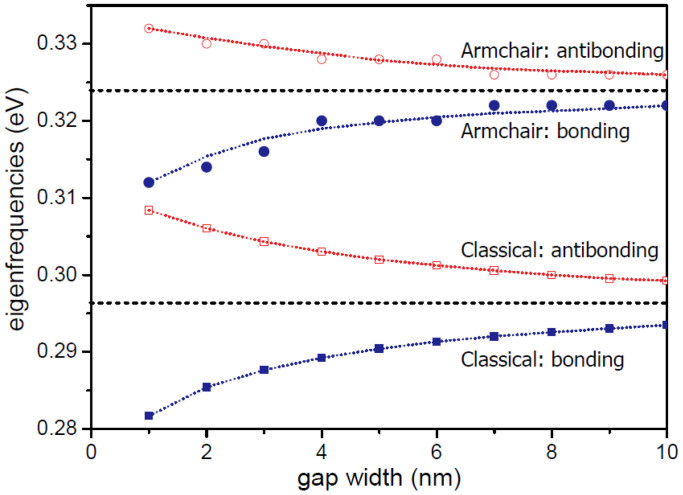
Gap dependence of hybridization in graphene dimers. The eigenfrequencies of the hybridized modes as a function of gap width for *x*-polarized dipoles in classical calculations and armchair triangles, respectively. The two dotted lines (0.296 eV and 0.324 eV) are the dipole eigenfrequencies associated with the individual triangles.

The energy splitting or coupling strength depends on the gap width of the bow-tie structures, which can be investigated in the hybridization of *x*-polarized dipoles. We calculate the eigenfrequencies of the hybridized plasmon modes as a function of the width gap, and show the results in Figure 6. The modes in zigzag triangles exhibit very small energy splitting, so we do not show them here. Both in the classical calculations and armchair-terminated triangles, the energy splitting decreases as the gap width increases. The decrease is most pronounced for gap widths below 4 nm, while the variation is weaker for larger separations.

**Figure 7:**
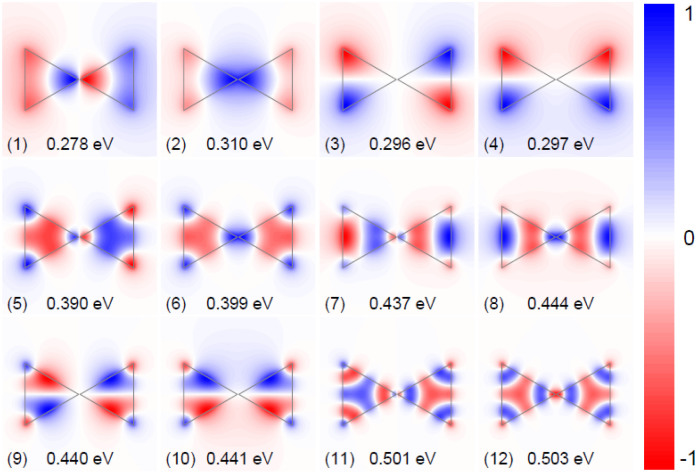
In-plane classical eigenmode potentials of graphene dimers. The real part of *φ_n_* for selected twelve plasmon modes calculated from the eigenvectors of the matrix pair BA of Eq. (3).

We note that the hybridization of other dimer plasmon modes (other than dipole modes) can be analyzed with a similar result. Generally speaking, the eigenfrequencies of the resulting hybridized modes are decided by two factors: symmetry and coupling strength. Specifically, the antisymmetrically coupled modes (no matter which polarization) have lower energy and modes with less field concentration at the gap region cause weaker coupling and consequently exhibit smaller energy splitting. As a further evidence for this qualitative characterization, we show in Figure 7 the selected twelve plasmon modes from classical calculations, corresponding to the peaks shown in Figure 4c. As compared with Figure 2, they can be understood as linear combinations of the wave patterns in individual structures. Likewise, it is straightforward to envision the wave patterns in armchair and zigzag bow-tie triangles based on the uncoupled modes from Figure 3.

## Discussion

In this article, we have considered and compared classical and quantum aspects of plasmonic eigenmodes in graphene triangular nanostructures. The 2D FEM-approach for calculation of the classical electromagnetic response represents a numerically highly efficient method for electrodynamics in general 2D morphologies of graphene structures in the electrostatic limit (see Supplementary Information for the calculation in hexagonal structures). The simple eigenvalue approach offers a direct pathway to extraction of all plasmonic eigenmodes, not limited to just the optically active, but including also dark modes and highly symmetric breathing modes. The quantum method adopted here is useful for investigating the quantum effects in plasmon excitations of smaller graphene structures, and it offers additional insight into the importance of the particular edge-termination of the underlying atomic lattice. By a sweep of the excitation energy, our calculation of the eigenvalue loss spectra enables direct identification of all plasmonic modes also in the quantum treatment.

We have applied both methods to equilateral triangles, of 20 nm sidelength, both in isolated and in bow-tie configurations. For the isolated nanotriangle we find that the plasmonic response of armchair-terminated triangles is qualitatively similar to the classical case, albeit with a significant and consistent blueshift of all resonances due to nonlocal response. Conversely, the response of zigzag-terminated triangles exhibits several significant differences from its classical counterpart. As a consequence of the existence of localized electronic edge states near zigzag edges, the eigenmodes extend further into the bulk, and are less intense at the vertices. Additionally, we observe a redshift and an pronounced readjustment of the loss-function intensity relative to the classical case.

In the bow-tie configuration we observe plasmon hybridization and associated eigenmode energy splitting, of varying degree depending on treatment; the largest splitting is observed in the classical approach, and the smallest in zigzag structures. Nevertheless, the effects of hybridization are qualitatively similar across the considered cases, with the antisymmetric hybridized modes exhibiting a lowered energy, and with the coupling strength - and associated energy splitting - decreasing when the constituent eigenmodes exhibit lower field intensities in the gap region.

## Methods

### Classical Calculations

The classical calculations are performed on the two-dimensional domain defined by the geometry of the graphene structure. The domain is discretized using a triangular mesh (see Supplementary Information for details), consisting of a set of elements 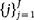 delimited by a set of vertices 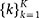. For future reference, we denote the region of the *j*th element by Ω*_j_*. With a sufficiently dense mesh, a faithful approximation of the Coulomb and Laplacian operators in Eqs. (1) and (2) can then be achieved with the approach described in the following. Crucially, this allows the reduction of the coupled integro-differential equations into simple algebraic equations, as summarized in Eq. (3).

To proceed, we introduce the following notation: the vertices of the *j*th element are denoted as *α_j_*, *β_j_*, and *δ_j_*, and together define the element centroid coordinates, 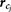 and values 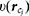 (representing e.g. the density)
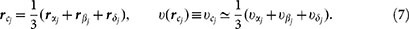


The integration of Eq. (1) can then be approximated directly by a Riemann sum at the centroids. With an aim to ultimately interlink the potentials, *φ_k_*, and the densities, *ρ_k_*, at all vertex positions 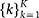, we can then evaluate Eq. (1) at the *k*th vertex to find
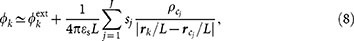
where we have introduced the area of Ω*_j_* as *s_j_*. Note that no explicit handling of a semi-divergent self-interaction is necessary since 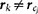 for all *j*. The matrix **A** can then be assembled by noting its equivalent definition in element form, 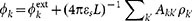, which upon comparison with Eq. (8) allows the identification 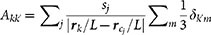, with the *m*-sum running over the vertex sites of Ω*_j_*, i.e. *m* ∈ {*α_j_*,*β_j_*,*δ_j_*}, and where the twice-subscripted *δ* denotes the Kronecker delta.

Rather than assembling the matrix **B** (the discretized representation of the Laplacian) from Eq. (2) directly, we identify it by its weak form, applying the ideas behind FEM, allowing us to enforce the boundary condition 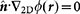 explicitly. Specifically, multiplying onto Eq. (2) an unspecified linear test function 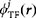, and integrating over the domain, we find
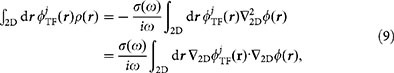
where the boundary condition 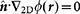 has been applied at the second equality sign. Next, we specify the linear test function 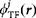. Concretely, we take the test function as nonzero only on Ω*_j_*. The value of the test function within Ω*_j_* is most conveniently specified in a local barycentric coordinate system with parameters *η* ∈ [0,1] and 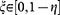, within which 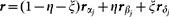 for 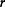 ∈ Ω*_j_*. Within each region Ω*_j_* we interpolate function values 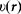, e.g. potentials or densities, according to their associated vertex values
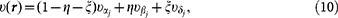
such that 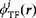 equals the approximated *φ*(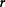) for 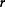 ∈ Ω*_j_*. Applying Eq. (10) to the left-hand side of Eq. (9) then yields
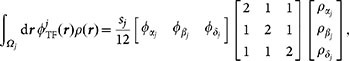


We can recast this result in terms of the full vertex vectors ***φ*** and ***ρ*** as equaling *φ^T^***B***^Lj^****ρ***, where **B***^Lj^* denotes a *K* × *K* matrix defined by 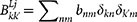 with the *nm*-sum restricted to {*n,m*} ∈ {*α_j_*, *β_j_*, *δ_j_*}, with nonzero elements defined by a 3 × 3 block matrix 
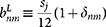
.

To evaluate the right-hand side of Eq. (9) we require an expression for 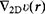 for 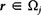, which can be obtained from Eq. (10) using straight-forward algebra, yielding
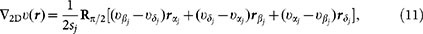
where 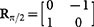 denotes a *π*/*2* counterclockwise rotation. Using this, the right-hand side integral in Eq. (9) becomes 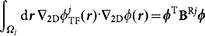 with **B**^R*j*^ denoting a *K* × *K* matrix defined similarly to **B**^L*j*^, i.e. as 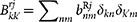, but with a *j*-dependent 3 × 3 block matrix
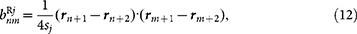
where the subscript-notation *n* + 1 and *n* + 2 indicates forward-cycling by 1 and 2, respectively, in the set {*α_j_*, *β_j_*, *δ_j_*} (e.g. if *n* = *β_j_* then *n* + 1 = *δ_j_* and *n* + 2 = *α_j_*, and equivalently for *m*).

Finally, by summing over all *j*, and defining **B**^L^ ≡ Σ*_j_***B**^L*j*^ and **B**^R^ ≡ Σ*_j_***B**^R*j*^, while noting that the test functions 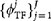 constitute a complete basis in the FEM sense, we can identify the weak form of Eq. (9) as **B**^L^***ρ*** = −*iσ*(*ω*)*ω*^−1^**B**^R^***φ***, from which we identify the desired matrix **B** with the property ***ρ*** = *iσ*(*ω*)*ω*^−1^**B*φ*** as **B** = −(**B**^L^)^−1^**B**^R^.

The classical material response is modeled by the bulk conductivity *σ*(*ω*) of graphene through its well-known local-response form^63^,^64^
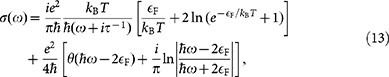
with the first and second terms due to intra- and interband dynamics, respectively. Here, *e* denotes the electron charge, *θ*(*x*) the Heaviside function, and ln(*x*) the natural logarithm.

### Quantum Calculations

The tight-binding Hamiltonian for the *π*-electrons is constructed by considering only nearest-neighbor interactions with a hopping strength *t* = 2.8 eV. The associated Hamiltonian matrix-representation is real-valued and symmetric, giving rise to real eigenvalues and eigenvectors.

The direct evaluation of the noninteracting density response matrix 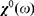 of Eq. (4) requires significant computational resources and time, amounting to ~*N*^4^ operations, which must additionally be repeated for each distinct frequency. Significant reduction of computational complexity, to ~*N*^3^, can be achieved with the aid of Hilbert and fast Fourier transforms (FFT), following a procedure developed in density-functional theory (DFT)^65^,^66^, and recently implemented in Ref. 40 for the tight-binding model of graphene considered here. We adopt the same technique in our computations.

Furthermore, consideration of the symmetry of 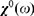, i.e. 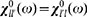, leads to an additional reduction of the computational requirements.

## Supplementary Material

Supplementary Information Supplementary Information
