# Supplementary material for: Plasmonic eigenmodes in individual and bow-tie graphene nanotriangles
Source: Sci Rep. 2015 Apr 9;5:9535. doi: 10.1038/srep09535 (PMC5396075; doi:10.1038/srep09535)
Supplement: Supplementary Information [file srep09535-s1.pdf]

Plasmonic eigenmodes in individual and bow-tie graphene nanotriangles

Weihua Wang,[†,‡] Thomas Christensen,[†,‡] Antti-Pekka Jauho,[¶,‡]
Kristian S. Thygesen,[§,‡] Martijn Wubs,[†,‡] and N. Asger Mortensen[*,†,‡]

DTU Fotonik, Center for Nanostructured Graphene, DTU Nanotech, and DTU Physics

E-mail: asger@mailaps.org

[*]To whom correspondence should be addressed
[†]Department of Photonics Engineering, Technical University of Denmark, DK-2800 Kgs. Lyngby, Denmark
[‡]Center for Nanostructured Graphene, Technical University of Denmark, DK-2800 Kgs. Lyngby, Denmark
[¶]Department of Micro- and Nanotechnology, Technical University of Denmark, DK-2800 Kgs. Lyngby, Denmark
[§]Department of Physics, Technical University of Denmark, DK-2800 Kgs. Lyngby, Denmark

2D Meshes in homogenous nanostructured graphene

We use the open source code Gmsh[1] to generate the triangular meshes employed in our classical calculations. In Figure S1 we show a simple example of such a mesh, extending a triangular graphene nanostructure. In the practical computations, we obtain satisfactory convergence and very good resolutions with about $K = 3000$ vertices (about $J = 6000$ elements). Due to the electrostatic scaling law,[2,3] a fixed mesh configuration can be used in the calculations of differently sized triangles. Regarding symmetry, we construct the bow-tie triangles by simply copying the mesh from one triangle to the mirrored triangle, allowing a potential speed up of convergence.

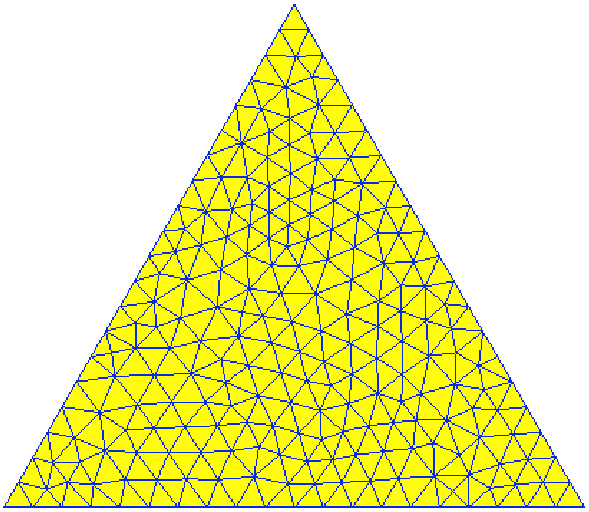

Figure 1: 2D meshes in a homogenous graphene triangle.

f_n in different nanostructures

The parameter $f_n = i\sigma(\omega_n)/(4\pi\varepsilon_s L\omega_n)$, introduced below Eq. (2), which gives a dimensionless number for the eigenresonances – independent of chosen conductivity profile – is solely dependent on the choice of geometry and characteristic feature length L. In Table 1 we list calculated $-f_n$ in different relevant geometries, indicating also the choice of L for each geometry.

Table 1: Tabulated dimensionless modal-eigenvalues, -f_n, listed in order of increasing energy, for different geometries as indicated. Doubly-degenerate eigenvalues are indicated explicitly by *. The choice of characteristic length L is indicated in red.

n					
1	0.0879*	0.0947*	0.1145*	0.1369*	0.1462*
2	0.0420	0.0689	0.0699*	0.0787*	0.0808*
3	0.0304*	0.0374	0.0397*	0.0637	0.0566*
4	0.0224*	0.0356	0.0386	0.0444	0.0464
5	0.0189	0.0349*	0.0356*	0.0437	0.0437*
6	0.0180	0.0233	0.0287	0.0400*	0.0358*
7	0.0154*	0.0230*	0.0248*	0.0359*	0.0326*
8	0.0139	0.0229	0.0231*	0.0311	0.0303*

The values of f_n in disks were studied in Ref. 6 using a highly accurate semi-analytical approach. For comparison, and to assess the accuracy of our numerical approach, we compare the value for the dipolar mode and find less than 1% relative deviation. Since the disk-results converge slower compared with the other geometries considered here (cf. the challenge of approximating a curved boundary with a triangular mesh), we expect the values in Table 1 to have less than roughly 1% relative deviation from exact values throughout.

Taking only the Drude term for the graphene surface conductivity [see Eq. (13) in main text], we have

$$-f_n = \frac{e^2}{4\pi\varepsilon_s L} \frac{\epsilon_{\mathrm{F}}}{\pi\hbar\omega_n(\hbar\omega_n + i\hbar\tau^{-1})}, \tag{S1}$$

illustrating that the plasmonic frequency scales as $\omega_n \mathrel{\overset{\propto}{\sim}} \sqrt{\epsilon_{\mathrm{F}}(\varepsilon_s L)^{-1}}$, indicating its tunability with Fermi energy ϵ_{F}, feature length L, and substrate properties via ε_s. Finally, the resonance line width is proportional to the relaxation loss $\hbar\tau^{-1}$ (associated with a finite carrier mobility).

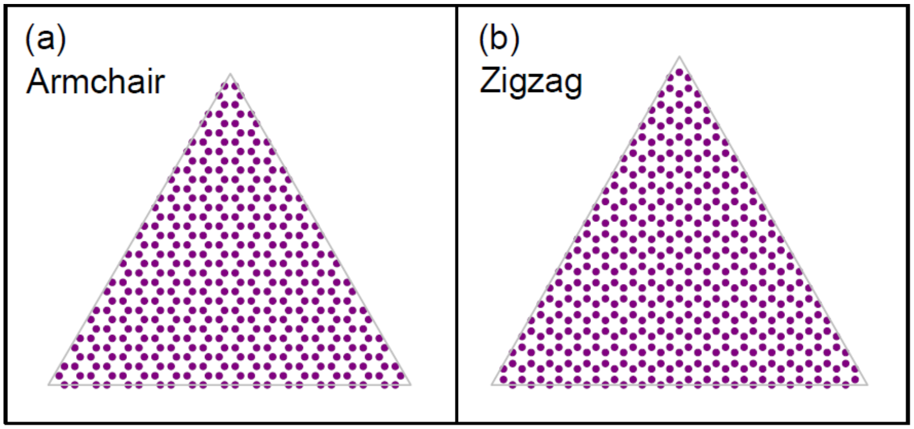

Figure 2: The atomic construction of armchair and zigzag triangles.

Construction of armchair and zigzag triangles

To construct the triangles with different edge terminations, we fix one of the vertices as the origin, arrange the carbon atoms in the bottom edge with different configurations, and fill the upper layers one by one with a decreasing width. Finally, we delete the single-bonded carbon atoms. Thus for a typical side length, the constructed triangles may not satisfy the rotation symmetry, but this can be realized by reducing or adding a few carbon atoms. We show in Figure S2 the nanotriangles with two different edge terminations.

Electronic edge states

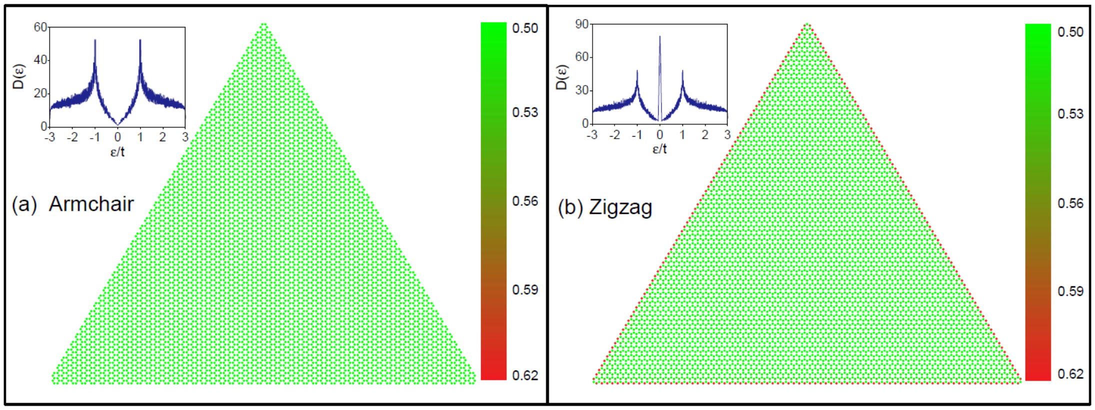

Figure 3: The local density of states at armchair (a) and zigzag triangles (b). Inset shows the density of states, where t denotes the hopping parameter.

The direct diagonalization of the tight-binding Hamiltonian matrix yields the single-particle energy states ϵ_j and the corresponding wave functions $\psi_j(\boldsymbol{r})$. The local density of states are computed through

$$n(\boldsymbol{r}) = \sum_j f_j \psi_j^*(\boldsymbol{r}) \psi_j(\boldsymbol{r}) \tag{S2}$$

with f_j the fermi distribution, and the energy density of states are

$$D(\epsilon) = -\frac{2\gamma}{\Delta} \mathrm{Im} \left[\sum_j \frac{1}{\epsilon - \epsilon_j + i\gamma} \right], \tag{S3}$$

where the factor 2 accounts for the spin degeneracy, γ is a very small damping factor (chosen as $\hbar\gamma = 0.006eV$ in the computation), and Δ is the surface area. The numerical results are shown in Figure S3, where we can see that the electrons are almost uniformly distributed on the structure with armchair edges, whereas non-uniform distributions are found for zigzag edges, with very large densities occurring at these edges. The consequence arises from an enormous amount of zero-energy states shown in the inset, which is essentially lacking in the structures with armchair edges.[4] These electronic edge states extremely affect the plasmon modes in both the resonance energy and damping.[5,6]

Calculations on graphene hexagons

The methods introduced in this article are very general for the calculations of different geometries. As an additional example, we will discuss the plasmon modes in graphene hexagons which are recently produced in experiments.[7,8] Using the parameters given in the main text, we show in Figure S4 the calculated eigenvalue loss spectrum, and similarly label relevant of the plasmon peaks with numerals. The corresponding 12 plasmon modes from classical calculations are plotted in Figure S5, where the first eight modes (1)-(8) are mirror symmetric and the last 4 modes (9)-(12) satisfy the same discrete rotational symmetry as the hexagonal structures. Due to the high symmetry properties of hexagonal structures, only the

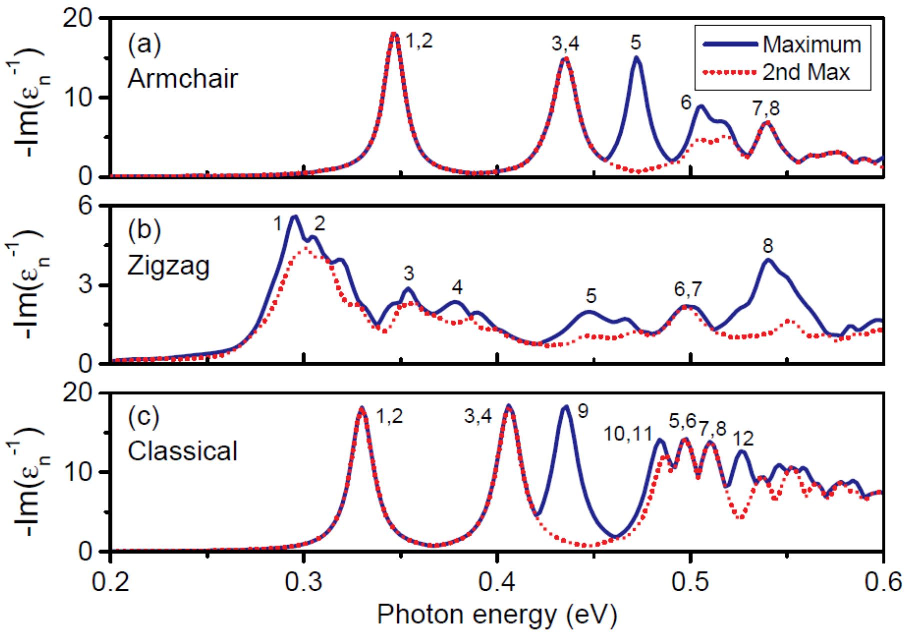

Figure 4: The eigenvalue loss spectrum $-\text{Im}(\varepsilon_n^{-1})$ in 10 nm sided armchair (a), zigzag (b), and homogenous (c) graphene hexagons.

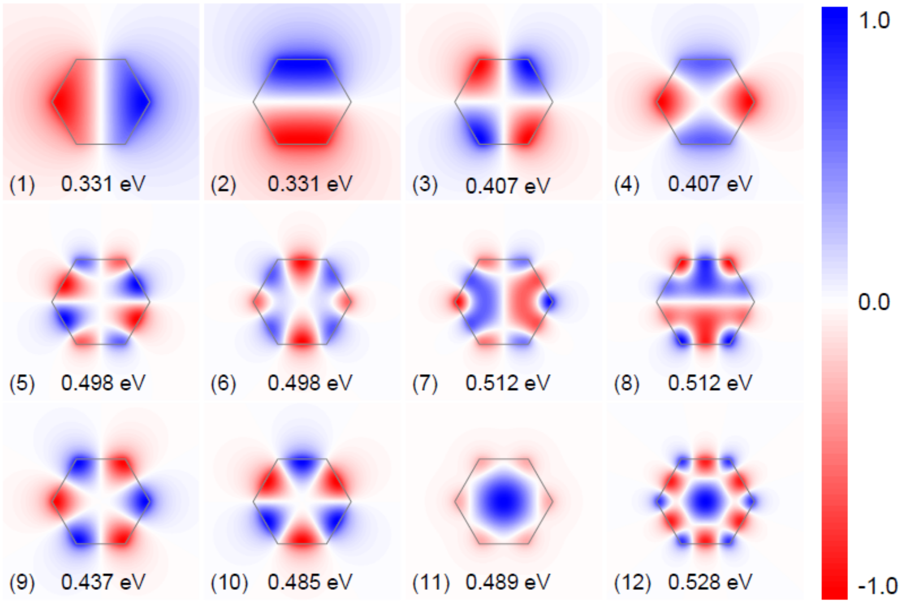

Figure 5: The 12 plasmon modes in a homogenous hexagon.

dipole modes (1)-(2) are optically active, and the other modes can only be detected by some near-field techniques, while in graphene triangles some of the multipole modes still have a net dipole moment. Modes (9) and (10) both exhibit discrete rotational symmetry. For a mirror that cuts through the middle of two opposite vertices, mode (9) has anti-symmetric

mirror symmetry and mode (10) is mirror symmetric.

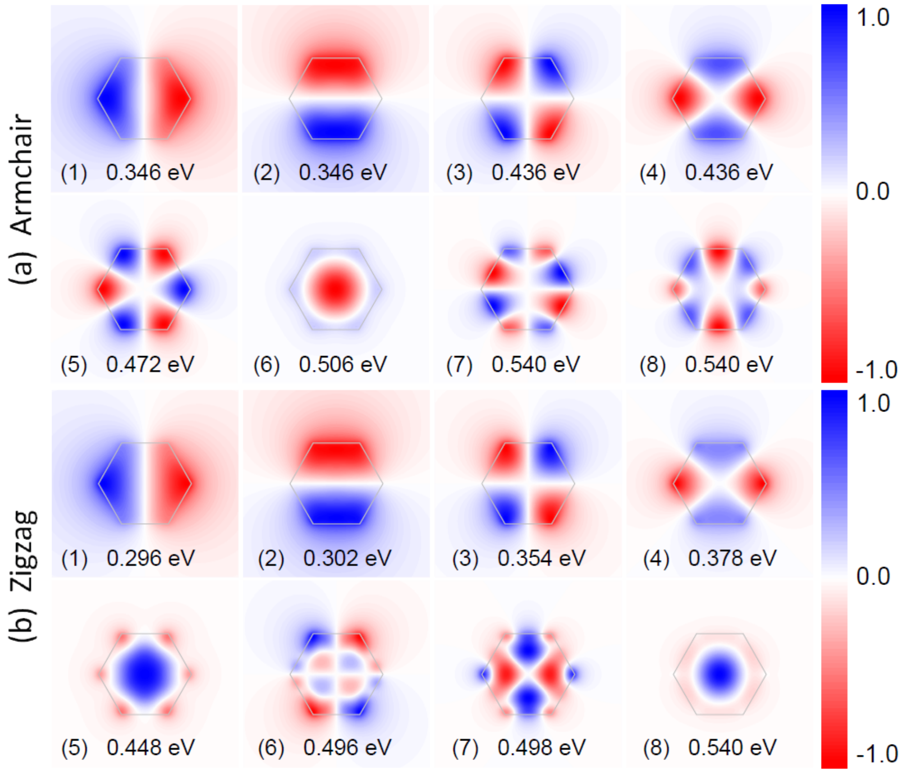

Figure 6: The selected 8 plasmon modes in hexagons with armchair (a) and zigzag (b) edges.

We also extract the mode patterns from the quantum calculations as shown in Figure S6. Similar to triangles, the plasmon modes exhibit blueshift in hexagons with armchair edges, but redshift in those with zigzag edges. The dipole modes are very similar in three cases, although due to the practical construction the two dipolar modes for two polarizations in zigzag hexagons have different energy. This may be due to the the relatively smaller perimeter to area ratio, being 3 times larger in triangles than that in hexagons. The differences in some higher order modes, for example the modes (7) and (8) in armchair hexagons and the modes (6) and (7) in zigzag hexagons, can be clearly observed.

We show in Figure S7 the eigenvalue loss spectrum in bow-tie graphene hexagons. The energy splitting is large in both homogenous and armchair hexagons bow-ties, also evident in a pronounced energy splitting for the y-polarized dipole modes, because of the relatively stronger coupling here which is almost absent in triangles. In zigzag hexagons, the cou-

pling is much weaker, and even the $x-$polarized dipole does not exhibit two significantly hybridized peaks. In Figure S7b, mode (1) (corresponding to the bonding dipole mode along $x-$direction) has an apparent peak, while the mode (2) (antibonding) is very close to modes (3) and (4) at current numerical precision. Except for the coupling strength, the hybridization procedure in bow-tie hexagons is then similar to those in bow-tie triangles. Figure 5 of the main text can thus be generalized to describe the plasmon hybridization in bow-tie graphene hexagons.

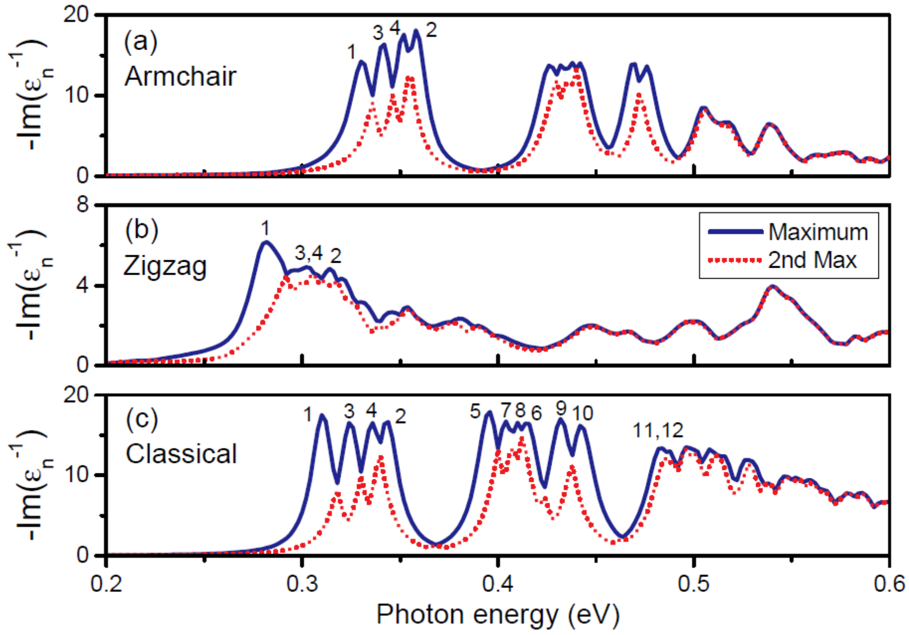

Figure 7: The eigenvalue loss spectrum $-\mathrm{Im}(\varepsilon_n^{-1})$ in bow-tie armchair (a), zigzag (b), and homogenous (c) graphene hexagons.

Finally, the 12 lowest-energy plasmon modes in bow-tie homogenous graphene hexagons are shown in Figure S8. In both armchair and zigzag bow-tie graphene hexagons, the mode patterns can be easily constructed from Figure S6, and thus will not be presented here.

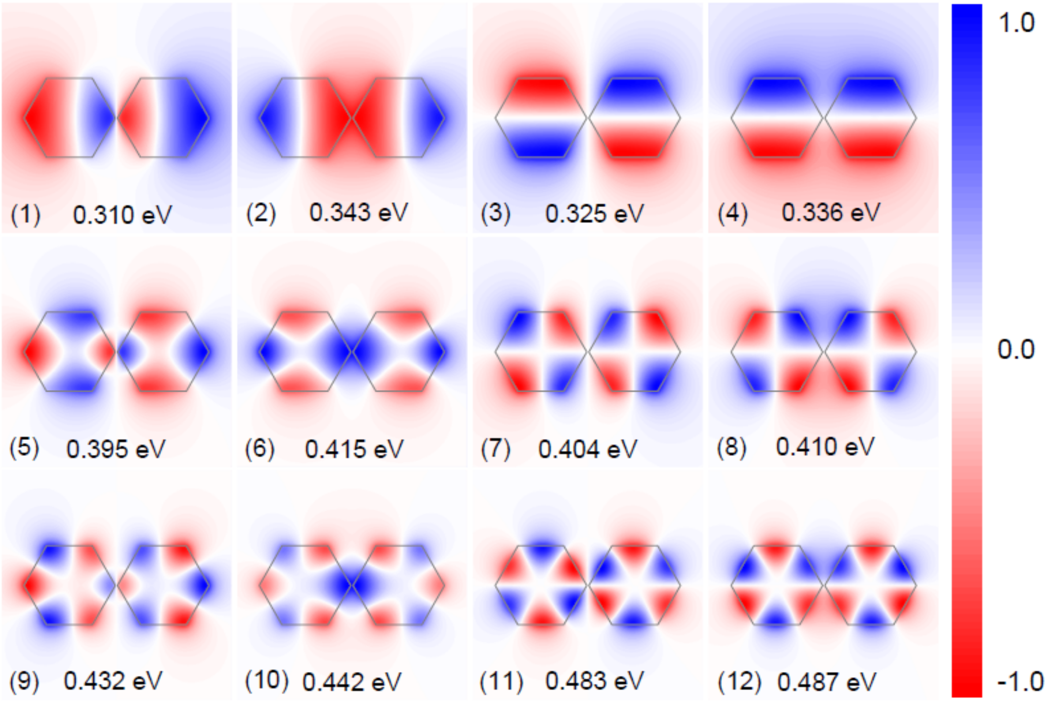

Figure 8: The 12 lowest energy plasmon modes in bow-tie homogenous graphene hexagons.

References

1. Geuzaine, C.; Remacle, J.-F. Gmsh: A 3-D finite element mesh generator with built-in pre- and post-processing facilities. *Int. J. Numer. Methods Eng.* **2009**, *79*, 1309–1331.

2. Christensen, J.; Manjavacas, A.; Thongrattanasiri, S.; Koppens, F. H. L.; García de Abajo, F. J. Graphene Plasmon Waveguiding and Hybridization in Individual and Paired Nanoribbons. *ACS Nano* **2011**, *6*, 431–440.

3. Wang, W.; Apell, S. P.; Kinaret, J. M. Edge Magnetoplasmons and the Optical Excitations in Graphene Disks. *Phys. Rev. B* **2012**, *86*, 125450.

4. Nakada, K.; Fujita, M.; Dresselhaus, G.; Dresselhaus, M. S. Edge state in graphene ribbons: Nanometer size effect and edge shape dependence. *Phys. Rev. B* **1996**, *54*, 17954–17961.

5. Thongrattanasiri, S.; Manjavacas, A.; García de Abajo, F. J. Quantum Finite-Size Effects in Graphene Plasmons. *ACS Nano* **2012**, *6*, 1766–1775.

6. Christensen, T.; Wang, W.; Jauho, A.-P.; Wubs, M.; Mortensen, N. A. Classical and Quantum Plasmonics in Graphene Nanodisks: the Role of Edge States. *Phys. Rev. B* **2014**, *90*, 241414(R).

7. Robertson, A. W.; Warner, J. H. Hexagonal Single Crystal Domains of Few-Layer Graphene on Copper Foils. *Nano Lett.* **2011**, *11*, 1182–1189.

8. Wu, B.; Geng, D.; Guo, Y.; Huang, L.; Xue, Y.; Zheng, J.; Chen, j.; Yu, G.; Liu, Y.; Jiang, L.; Hu, W. Equiangular Hexagon-Shape-Controlled Synthesis of Graphene on Copper Surface. *Adv. Mater.* **2011**, *23*, 3522–3525.
